# MEDICAL EXPENDITURE DIFFERENCES: EMERGING OLDER ADULTS, BOOMERS VERSUS THE PREDECESSORS IN KOREA (2010–2020)

**DOI:** 10.1093/geroni/igae098.1786

**Published:** 2024-12-31

**Authors:** Yejin Lim, Youngtae Cho, Jung-Hwa Ha, Woorim Ko

**Affiliations:** Seoul National University Graduate School of Public Health, Seoul, Republic of Korea; Seoul National University, Seoul, Republic of Korea; Seoul National University, Seoul, Republic of Korea; Seoul National University, Seoul, Republic of Korea

## Abstract

South Korea, the world’s fastest-aging society, faces growing concerns over the increasing societal caregiving burden as the aging of the baby boom generation (boomers) starting in 2020. Recognizing the importance of generational context in gerontological research, this study conducted APC analysis on the annual individual medical expenditure (AIME) of approximately 148,000 participants from the Korea Health Panel Survey data (2010-2020), comparing the industrial generation (IG) (born 1945-1954) with the boomers (born 1955-1964). The analysis revealed a declining AIME trend from the IG to the boomers, with age and period effects controlled. This trend was similarly observed in AIME related to out-patient (OP) services and services at primary care providers, contrasting with AIME related to emergency or in-patient services and care at higher-level institutions. The findings suggest that the difference in total AIME between the two generations could stem from a decrease in the use of OP services at primary healthcare institutions by the boomers. Further analysis, guided by the relationship between OP service use and the number of non-communicable diseases (NCDs) reported in prior studies, indicated that the boomers had a significantly lower incidence of major NCDs compared to the IG. This research highlights how the boomers, having driven Korea’s post-war economic boom, are shaping into a distinct older population, diverging from aging patterns of the predecessor generation. It underscores the importance of developing sustainable health management and expenditure policies that reflect the needs of emerging older adults.

